# From cellular heterogeneity to precision medicine: single-cell multi-omics in CNS disease research

**DOI:** 10.3389/fncel.2026.1848558

**Published:** 2026-06-11

**Authors:** Tingyu Liu, Yan Zhang, Wei Hou, Hui Hao, Anqi Geng, Gang Zhao, Yingying Zhang

**Affiliations:** 1 The College of Medicine, Northwest University, Xi'an, China; 2 Honghui Hospital, Xi’an Jiaotong University, Xi’an, China; 3 Research and Development Institute of Northwestern Polytechnical University in Shenzhen, Shenzhen, Guangdong, China; 4 The First Hospital of Northwest University, Xi'an, China

**Keywords:** cellular heterogeneity, central nervous system diseases, multi-omics, precision medicine, single-cell sequencing

## Abstract

Single-cell sequencing and multi-omics technologies are revolutionizing research on central nervous system (CNS) diseases by enabling high-resolution analysis of cellular heterogeneity and molecular dynamics. Traditional technologies (e.g., bulk sequencing, routine histology) often lack cellular resolution, fail to capture heterogeneity among individual cells, and struggle to reveal subtle molecular changes in early pathogenesis, limiting their ability to clarify complex CNS disease mechanisms and develop precise diagnostic tools. This review comprehensively summarizes the latest advances in single-cell multi-omics methodologies, including genomics, transcriptomics, proteomics, metabolomics, and spatial omics, and their applications in elucidating the pathogenesis, diagnosis, and treatment of common CNS disorders. Representative diseases such as ischemic stroke, Alzheimer’s disease, Parkinson’s disease, viral meningitis, bacterial meningitis, multiple sclerosis, autism spectrum disorder, and depression are used as examples to discuss the current status and future prospects of single-cell multi-omics technologies in CNS disease research. Currently, these technologies have enabled the identification of rare pathogenic cell subsets, the mapping of cell-specific molecular pathways, and the discovery of potential diagnostic biomarkers in several common CNS disorders, though their clinical translation is still hindered by technical costs and standardization issues. In the future, the integration of single-cell multi-omics with spatial transcriptomics, artificial intelligence, and clinical data is expected to further decode the complex pathogenesis of CNS disorders, accelerate the development of targeted therapies, and promote the shift toward personalized medicine in CNS disease management—aligning with translational goals of neuropsychopharmacology.

## 1 Introduction

The core challenges of CNS diseases lie in their pronounced cellular heterogeneity, the complex and dynamically changing tissue microenvironment, the multi-layered and intertwined molecular mechanisms, and the difficulty of directly obtaining brain tissue in human studies (Song et al., 2025). These diseases often involve the coordinated abnormalities of various cell types, including neurons, astrocytes, microglia and oligodendrocytes, which exhibit highly specific response patterns at different stages of the disease. Traditional bulk omics and conventional pathological approaches can only reflect overall averaged signals, often masking key cell type–specific changes, failing to resolve the roles of rare cell populations, and making it difficult to reconstruct complex intercellular interaction networks and dynamic processes (Stur et al., 2022). This limitation is particularly evident when studying early-stage or localized lesions, leading to many potential pathogenic mechanisms and key regulatory factors being overlooked. In contrast, single-cell multi-omics technologies enable precise characterization of the molecular features of different cell types at single-cell resolution, identify rare yet functionally critical cell populations, and, through integrating multi-dimensional datasets such as single-cell transcriptomics, proteomics, and spatial omics, reconstruct intercellular interaction networks and elucidate dynamic communication among cells within the microenvironment. This integrated analysis not only reveals the plasticity of cell states but also provides a new perspective for understanding disease progression (Yousuf et al., 2023; Liu et al., 2025). At the same time, spatial omics allow the molecular information to be mapped to its precise spatial context, and, combined with dynamic tracking capabilities, can more accurately capture the disease’s dynamic evolution (Bressan et al., 2023). Together, these advances enable a comprehensive integration of multi-level molecular regulatory mechanisms, from gene expression, epigenetic modifications to protein interactions, thoroughly dissecting the molecular network reconstruction under disease conditions.

### 1.1 Current status of single-cell multi-omics research

**Figure 1:**
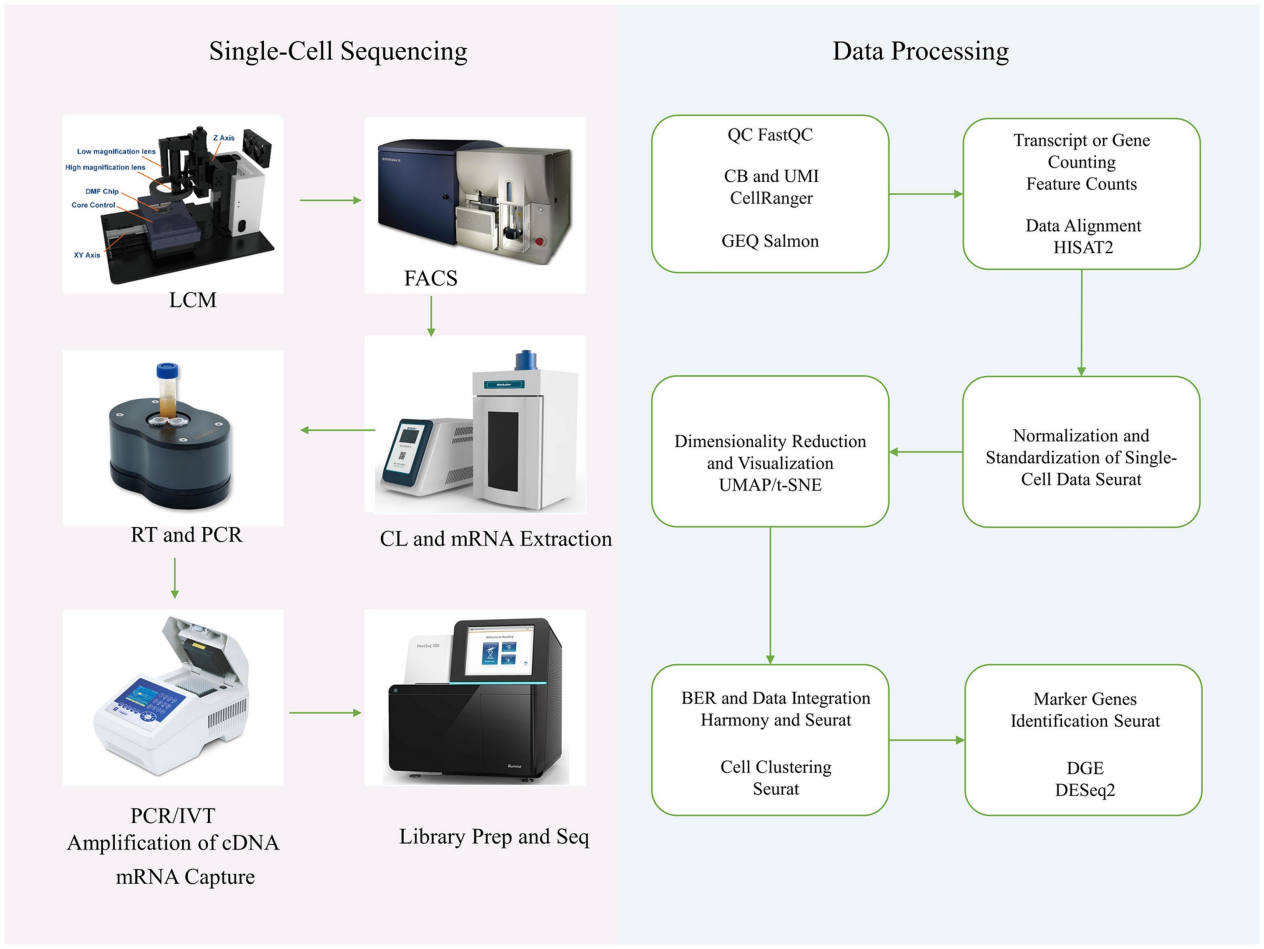
Methods of scRNA-Seq and its data processing workflow. First, single cells are precisely isolated via laser capture microdissection (LCM) or fluorescence-activated cell sorting (FACS). The isolated cells are then lysed to extract mRNA, which is reverse-transcribed (RT) and amplified by polymerase chain reaction (PCR) to generate cDNA. The cDNA is further amplified and enriched by PCR or *in vitro* transcription (IVT) before being constructed into sequencing libraries and sequenced on high-throughput platforms. Once raw sequencing data are obtained, quality control (QC) is performed using FastQC, followed by cell barcode (CB) and unique molecular identifier (UMI) processing using CellRanger. Next, gene expression quantification (GEQ) is conducted using Salmon, followed by transcript/gene counting and sequence alignment with tools such as HISAT2, to generate the gene expression matrix. The data are then normalized and standardized using Seurat, visualized via dimensionality reduction with Uniform Manifold Approximation and Projection (UMAP) or t-distributed Stochastic Neighbor Embedding (t-SNE). Batch effect correction (BER) and multi-batch data integration are performed using tools like Harmony for cell clustering. Finally, marker genes for each cell cluster are identified using Seurat, and differential gene expression (DGE) analysis is conducted with Differential Expression Analysis for Sequence Count Data 2 (DESeq2), providing critical evidence for subsequent biological interpretation.

At present, among existing cell sequencing technologies, single-cell RNA sequencing (scRNA-seq) technology is the most widely used in scientific research (Lei et al., 2021). The process of scRNA-seq includes the following steps (Figure 1). Briefly, single cells are isolated by laser capture microdissection (LCM) or fluorescence-activated cell sorting (FACS), followed by mRNA extraction, reverse transcription, and amplification to generate cDNA. After library preparation and high-throughput sequencing, raw data are processed to construct a gene expression matrix. The matrix is then normalized, visualized, and integrated across batches for cell clustering, marker gene identification, and differential gene expression analysis, which lays a foundation for downstream biological analysis.

**Figure 2:**
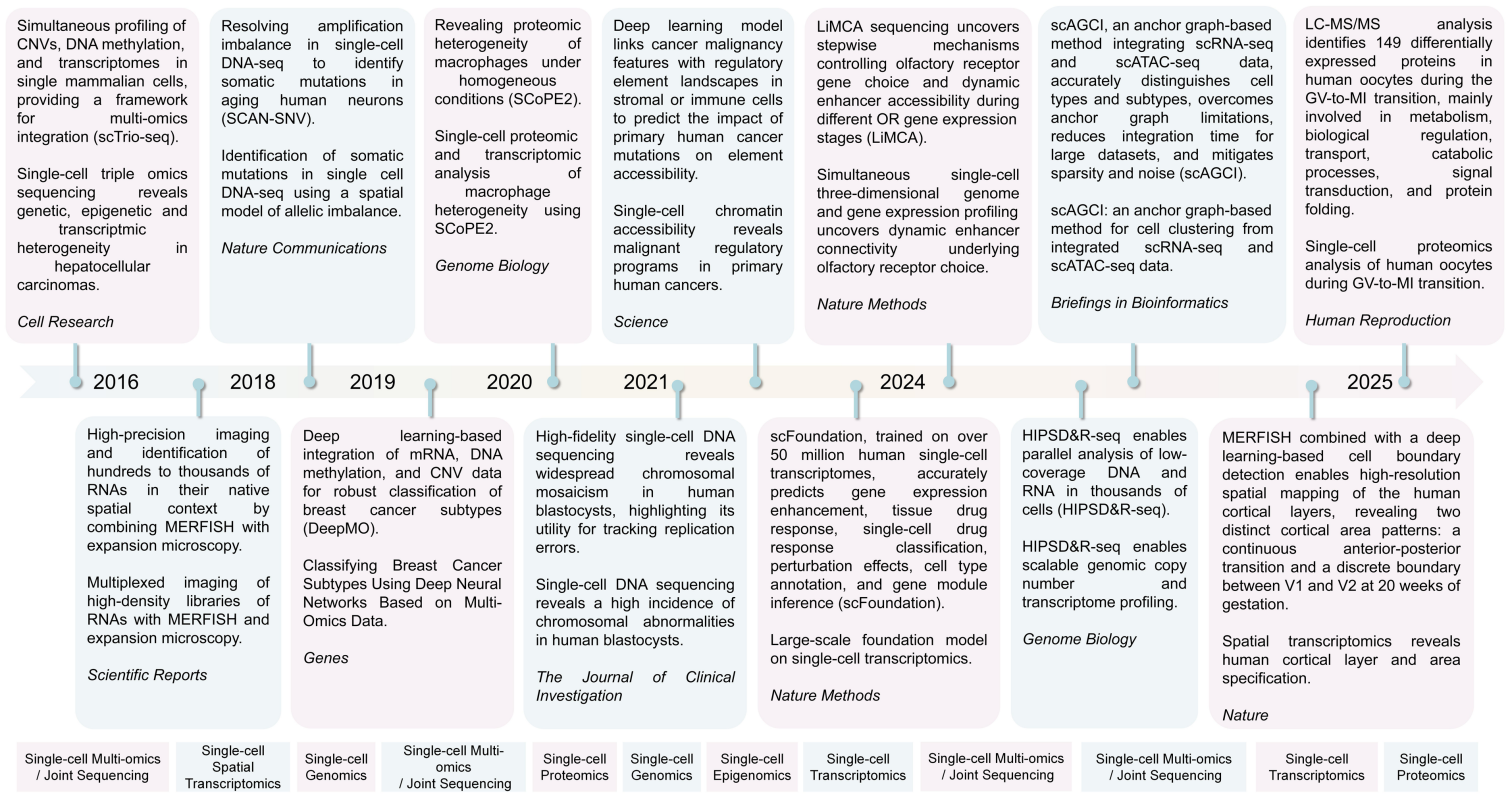
A timeline of key applications of single-cell multi-omics in deciphering CNS pathologies over the past decade. The bottom row below the timeline provides one-to-one technical category labels for each milestone study listed above. Each label classifies the corresponding work into a specific single-cell omics technology category. Repeated labels indicate that multiple milestone studies belong to the same technical category, thereby illustrating the evolving trends of each single-cell omics direction over time. ScTrio-seq, single-cell triple-omics sequencing; SCAN-SNV, single-cell allelic imbalance detection for single nucleotide variant; SCoPE2, single-cell proteomics with efficient lysis and extraction 2; LiMCA, linking mRNA to chromatin architecture; scAGCI, anchor graph-based cell clustering integrating scRNA-seq and scATAC-seq; LC–MS/MS, liquid chromatography–tandem mass spectrometry; MERFISH, multiplexed error-robust fluorescence *in situ* hybridization; DeepMO, deep multi-omics model; scFoundation, single-cell foundation model; HIPSD&R-seq, high-throughput single-cell DNA and RNA sequencing; V1, primary visual cortex; V2, secondary visual cortex.

In 2019, single-cell multi-omics was selected as the “Method of the Year” by Nature Methods (Figure 2) (Nature Methods, 2020). In the field of single-cell genomics, the Single-Cell Allelic Imbalance detection for Single Nucleotide Variant (SCAN-SNV) framework developed by Luquette et al. (2019) solved the problem of imbalanced amplification in gene detection. Chavli et al. (2024) utilized the high fidelity of single-cell genomics to discover the widespread presence of chromosomal mosaicism in human blastocysts, highlighting the advantage of single-cell genomics in tracking replication errors of genetic material.

For single-cell epigenomics, Lin et al. (2020) mined key features of breast cancer subtypes using a three-omics dataset (including mRNA data, DNA methylation data, and copy number variation data) and the deep learning model DeepMO. Sundaram et al. (2024) employed Assay for Transposase-Accessible Chromatin using Sequencing (ATAC-seq) to map the chromatin accessibility profile of cancer cells and construct a model for predicting the impact of mutations.

Regarding single-cell transcriptomics, the single-cell foundation (scFoundation) model developed by Hao et al. (2024) was trained on more than 50 million human single-cell transcriptomes and exhibited excellent performance in drug response prediction, cell type annotation, and other tasks.

In terms of single-cell proteomics, the Single-Cell Proteomics with Efficient Lysis and Extraction 2 (SCoPE2) method developed by Specht et al. (2021) revealed the proteomic heterogeneity of macrophages. Zhang et al. (2025) identified 149 differential proteins in human oocytes during the meiotic transition stage; these proteins are mainly involved in biological processes such as cellular metabolism and biological regulation, and related to pathways including signal transduction and protein folding.

For single-cell spatial omics, among imaging-based technologies, as early as 2018, Wang et al. (2018) proposed combining Multiplexed Error-Robust Fluorescence *In Situ* Hybridization (MERFISH) with expansion microscopy to achieve high-precision RNA imaging. For sequencing-based technologies, the 10x Genomics Visium platform can reconstruct the spatial structure of cells. Qian et al. (2025) further mapped a single-cell resolution spatial atlas of the human cerebral cortex using MERFISH and deep learning algorithms, identifying two distinct cortical regional patterns: first, a continuous and gradual transition of most cortical regions along the anteroposterior axis; second, a discrete and abrupt boundary between the primary visual cortex (V1) and secondary visual cortex (V2) visual cortices specifically identified at 20 weeks of gestation.

In terms of the joint analysis of single-cell multi-omics data and technological integration, researchers are also continuously exploring more efficient integration strategies. As early as 2016, Hou et al. (2016) published the single-cell triple-omics sequencing technology (scTrio-seq), which can simultaneously analyze copy number variations (CNVs), DNA methylation, and transcriptome in a single mammalian cell, providing ideas for upstream data integration. Wu H. et al. (2024) developed a sequencing method called “Linking mRNA to Chromatin Architecture” (LiMCA), revealing the determination mechanism of olfactory receptor (OR) genes and the dynamic changes in OR enhancer accessibility. Otoničar et al. (2024) proposed High-Throughput Single-Cell DNA and RNA Sequencing (HIPSD&R-seq), which enables parallel analysis of low-coverage DNA and RNA in thousands of cells based on improvements to the scATAC and multi-omics analysis of the 10x Genomics platform.

Meanwhile, innovations in analytical methods also provide key support for the in-depth mining of multi-omics data. Athaya et al. (2023) summarized the application of Multimodal Deep Learning (MDL) in single-cell multi-omics, compiling 21 non-horizontal integration cases and classifying these models into seven categories, including Variational Autoencoder (VAE), Autoencoder (AE), Generative Adversarial Network (GAN), Fully Connected Neural Network (FCNN), Graph Neural Network (GNN), and heterogeneous models (Argelaguet et al., 2021). They also pointed out that the main challenges currently faced by MDL include data preprocessing, requirements for data information, insufficient interpretability of modality-specific information in joint latent embeddings, and selection of hyperparameters, which provides insights for future innovations in analytical methods. Dong et al. (2025) proposed an anchor graph-based cell clustering method integrating scRNA-seq and scATAC-seq data, termed scAGCI. scAGCI performs excellently in distinguishing different cell types and subtypes, overcoming the limitations of anchor graphs in omics data characterization, reducing the integration time of large-scale datasets, and decreasing the sparsity and noise of omics data. In summary, single-cell multi-omics technology has achieved a leap from the conceptual proposal to practical implementation, realizing a transition from single-dimensional omics analysis to multimodal data integration. This advancement is propelling medicine toward greater precision and systematic understanding, laying a solid foundation for solving more biological problems and promoting the development of precision medicine.

## 2 Current status of multi-omics research

As a systems biology approach that integrates multi-dimensional omics data, multi-omics has continued to innovate on the basis of mature single-omics technologies (Baysoy et al., 2023; Vandereyken et al., 2023). Currently, long-read sequencing technology in genomics—capable of sequencing human DNA fragments ranging from 10,000 to 100,000 base pairs—has been applied in the research of neurodegenerative diseases (Su et al., 2021). In proteomics, the data-independent acquisition strategy combined with trapped ion mobility time-of-flight (timsTOF) mass spectrometry has improved detection efficiency and coverage (Lou and Shui, 2024; Vitko et al., 2024). For transcriptomics, the NanoString nCounter technology and reverse transcription-quantitative polymerase chain reaction technology each have their own advantages (Zucha et al., 2021; Class et al., 2023). The Single-Cell Energy Metabolism Profiling (SCENITH) technology in metabolomics enables the analysis of single-cell energy metabolism, while spatial omics achieves subcellular resolution research with the help of advanced microscopic technologies such as super-resolution microscopy (Schermelleh et al., 2019; Argüello et al., 2020). Beyond the development of more advanced single-omics sequencing methods, a key current direction in multi-omics research lies in better integrating multi-modal data generated by different omics technologies—for instance, by introducing algorithms like Pattern Fusion Analysis (PFA) and remapping to enhance technical performance (Shi et al., 2017). In 2023, Sathyanarayanan et al. (2023) proposed two core integration strategies: multi-stage integration and meta-dimensional integration. Multi-stage integration analyzes associations between different omics datasets in a stepwise manner, facilitating causal research; meta-dimensional integration, by contrast, analyzes multiple omics data simultaneously and requires feature selection to reduce computational load. These two strategies provide a direction for the efficient integration of multi-modal data.

In the field of applications, Sun et al. (2023) combined spatial metabolomics, lipidomics, and transcriptomics to identify cell types in the tumor microenvironment and “tumor-normal interface” regions, aiding in the understanding of cancer metabolism. Tao et al. (2023) analyzed genomic, epigenomic, and transcriptomic data from patients with colorectal cancer and gastric adenocarcinoma, and established a relevant scoring system to evaluate cancer staging and survival time. Ravi et al. (2022) characterized glioblastoma using spatially resolved transcriptomics, metabolomics, and proteomics, revealing tumor-host interdependencies. Jiang et al. (2020) developed Single-Cell RNA-seq Database for Alzheimer’s disease (scRead), a database of scRNA-seq and single-nucleus RNA sequencing (snRNA-seq) datasets for Alzheimer’s disease, which provides results of multi-regional data analysis. Pan et al. (2023) launched Ursa, a multi-omics toolkit that integrates scRNA-seq, single-nucleus Assay for Transposase-Accessible Chromatin using Sequencing (scATAC-seq), spatial transcriptomics, single-cell copy number variation (scCNV) analysis, scImmune analysis, Cytometry by Time-of-Flight (CyTOF), and flow cytometry, supporting integrated data analysis across multiple technologies. It is evident that multi-omics technology provides new insights for disease diagnosis and treatment, helps unravel the mechanisms of tumors and neurological diseases, and drives the development of precision medicine. To systematically compare the technical characteristics and application values of different single-cell multi-omics technologies in CNS disease research, we have summarized their core technical representatives, research focuses, CNS-specific applications and advantages in Table 1.

**Table 1:** Comparison of single-cell and multi-omics technologies for CNS disease research.

Omics type	Key representative technologies	Core research value in CNS diseases	CNS-related application examples	Main advantages
Single-cell genomics	SCAN-SNV framework	Resolve genetic heterogeneity of neural cells and track replication errors of genetic material	Genetic evolution of glioblastoma, pathogenic mutation analysis of epilepsy, somatic variation studies of neurodegenerative diseases	High-fidelity detection of DNA sequence variations and chromosomal mosaicism at single-cell resolution
Single-cell epigenomics	ATAC-seq, DeepMO deep learning model	Reveal epigenetic regulatory mechanisms of neuroinflammation and neuronal degeneration	Histone acetylation regulation in Alzheimer’s disease, chromatin remodeling in multiple sclerosis, epigenetic abnormalities in neurodevelopmental disorders	Capture genome-wide regulatory landscapes without altering DNA sequences
Single-cell transcriptomics	scFoundation large model	Identify cell subtypes in the CNS microenvironment and map cell-specific gene expression profiles	Dissection of cellular heterogeneity in ischemic stroke, Alzheimer’s disease, Parkinson’s disease, viral/bacterial meningitis, and depression	Highest throughput, most mature analytical pipeline, and the most widely used technology in current CNS research
Single-cell proteomics	SCoPE2 technology	Directly detect functional protein expression and post-translational modifications in neural cells	Validation of CNS disease biomarkers, functional confirmation of therapeutic targets, and functional phenotype analysis of neural cells	Directly corresponds to the actual functional state of cells and serves as a bridge connecting transcriptome and phenotype
Single-cell spatial omics	MERFISH, 10x Genomics Visium	Preserve the in situ spatial structure of brain tissue and analyze cell spatial distribution and local interactions	Human cerebral cortex spatial atlas mapping, neural circuit remodeling in epilepsy, and spatial heterogeneity studies of glioma microenvironment	First achieve integration of molecular expression information and tissue anatomical localization
Multi-omics integration technology	scTrio-seq, LiMCA, HIPSD&R-seq, scAGCI, MDL	Integrate multi-dimensional molecular data and systematically dissect complex regulatory networks of CNS diseases	Bidirectional tumor-host interdependencies in glioblastoma, construction of Alzheimer’s disease multi-omics database (scRead), and development of cross-platform data analysis tool (Ursa)	Break through the limitations of single omics and comprehensively reveal disease molecular mechanisms

SCAN-SNV, Single-Cell Allelic Imbalance detection for Single Nucleotide Variant; ATAC-seq, Assay for Transposase-Accessible Chromatin using Sequencing; DeepMO, Deep Multi-Omics model; scFoundation, single-cell foundation model; SCoPE2, Single-Cell Proteomics with Efficient Lysis and Extraction 2; MERFISH, Multiplexed Error-Robust Fluorescence In Situ Hybridization; scTrio-seq, single-cell triple-omics sequencing; LiMCA, Linking mRNA to Chromatin Architecture; HIPSD&R-seq, High-Throughput Single-Cell DNA and RNA Sequencing; scAGCI, anchor graph-based cell clustering integrating scRNA-seq and scATAC-seq; MDL, Multimodal Deep Learning; scRead, Single-Cell RNA-seq Database for Alzheimer’s Disease.

## 3 Central nervous system diseases

CNS primarily consists of the brain and spinal cord, which are protected by the skull, vertebral column, meninges, and the blood–brain barrier. As the most sophisticated and vital regulatory center of the human body, the CNS dominates neural signal transmission, sensory information integration, voluntary and involuntary motor control, advanced cognitive activities such as learning, memory and thinking, emotion modulation, neuroendocrine regulation, and systemic physiological homeostasis maintenance. It comprises highly heterogeneous cell types, including neurons, astrocytes, microglia, oligodendrocytes, and other glial cells, which collaboratively construct complex neural circuits and microenvironments to sustain normal physiological functions (Zong et al., 2023; Salvador et al., 2024).

CNS diseases represent a broad category of complex pathological conditions that disrupt the anatomical integrity, cellular homeostasis, and physiological function of the brain and spinal cord. These disorders can be triggered by multiple endogenous and exogenous risk factors, including genetic susceptibility, congenital developmental abnormalities, pathogenic microbial infection, age-related progressive neurodegeneration, physical trauma, immune inflammatory dysregulation, metabolic disorders, and environmental toxic stimulation. Such pathological insults ultimately lead to irreversible neuronal loss, glial cell activation and dysfunction, neural circuit disruption, synaptic plasticity impairment, and abnormal inflammatory responses, subsequently giving rise to diverse neurological, psychiatric, and behavioral clinical symptoms (Zyck et al., 2020; Fan and Huo, 2021).

The high incidence, high disability rate, and high mortality rate of CNS diseases are indisputable facts. Data from 2021 demonstrate that these heterogeneous disorders affect approximately 43% of the global population, equivalent to 3.4 billion individuals. Regarding high disability rates, diseases such as multiple sclerosis (MS) can cause physical impairments, psychological changes, and social dysfunction (Xu et al., 2019). Concerning high mortality rates, bacterial meningitis can have a fatality rate as high as 50%; with appropriate antibiotic treatment, mortality drops to 5–20% (Zuhorn et al., 2021). Meningitis caused by herpesvirus, if untreated, has a mortality rate up to 70%; even with antiviral treatment, mortality remains around 20–30% (Han et al., 2022). Moreover, CNS infections caused by fungi and parasites are clinically intractable, with mortality rates ranging from 30 to 50% (Zografou et al., 2021). Collectively, CNS diseases have become a leading global cause of compromised health status, lifelong disability, and premature death, contributing to approximately 443 million years of healthy life lost globally.

For systematic research and clinical therapeutic convenience, the present review categorizes CNS diseases into five core categories plus an additional “Others” section: (1) Cerebrovascular diseases: ischemic stroke (IS); (2) Neurodegenerative diseases: Alzheimer’s disease (AD) and Parkinson’s disease (PD); (3) CNS infectious diseases: bacterial meningitis (BM) and viral meningitis (VM); (4) CNS demyelinating diseases: multiple sclerosis; (5) Neurodevelopmental and psychiatric disorders: autism spectrum disorder (ASD) and depression (GBD 2021 Nervous System Disorders Collaborators, 2024).

**Figure 3:**
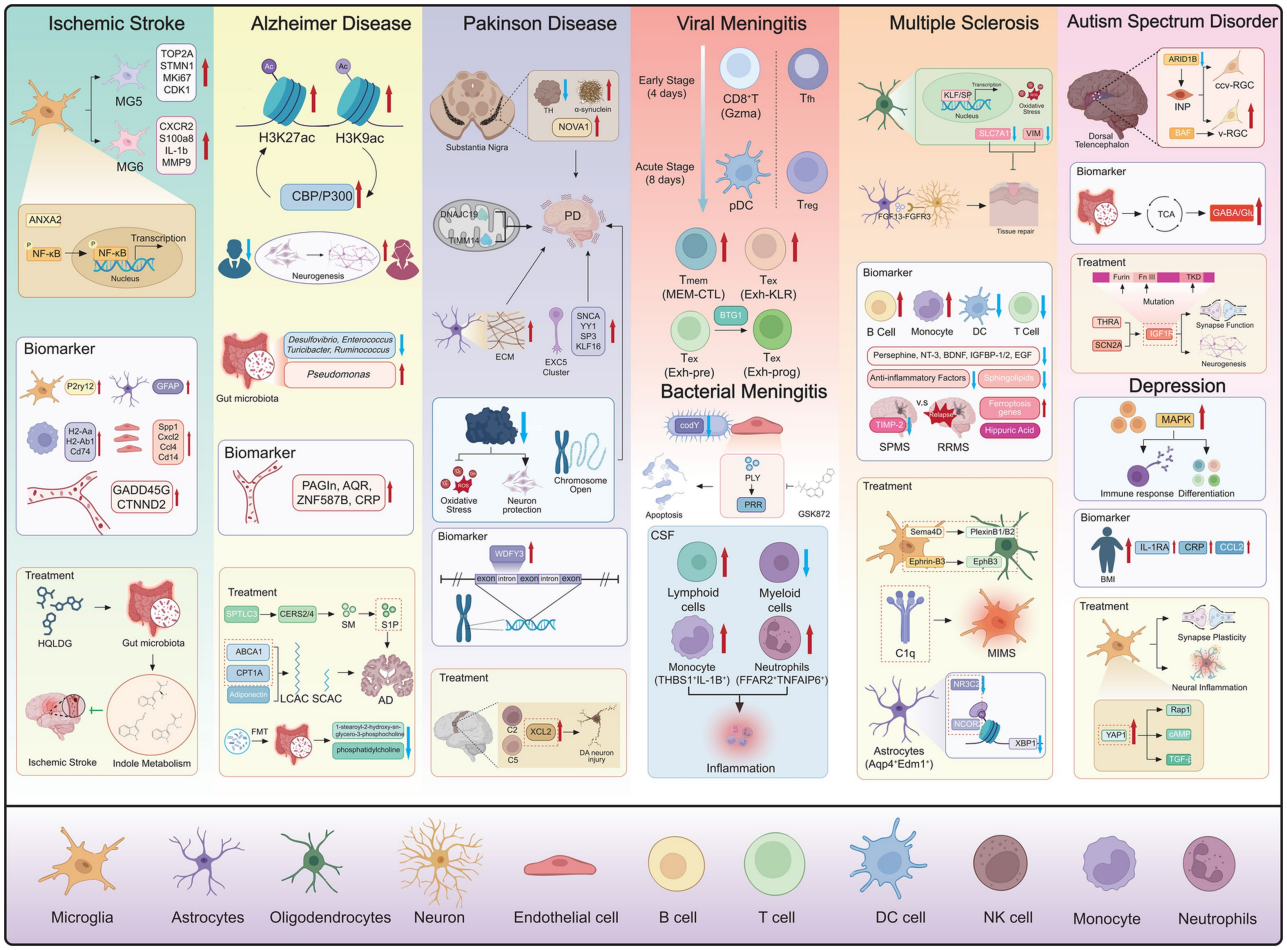
Diagram of new target pathway mechanisms for the CNS diseases discovered by single-cell multi-omics. For ischemic stroke, key pathways include microglial activation (MG5/MG6) and Annexin A2 (ANXA2)-mediated nuclear factor kappa-light-chain-enhancer of activated B cells (NF-κB) signaling, involving molecular targets such as DNA topoisomerase II alpha (TOP2A), stathmin 1 (STMN1), nuclear paraspeckle assembly transcript 1 (NEAT1), C-X-C motif chemokine receptor 2 (CXCR2), S100 calcium-binding protein A8 (S100a8), and L1 retrotransposon-derived macrophage inflammatory proteins (L1-MIPs), alongside biomarkers including purinergic receptor Y12 (P2ry12), glial fibrillary acidic protein (GFAP), aquaporin-4 autoantibody (HA-AQP4), S100 calcium-binding protein beta (S100β), C-C motif chemokine receptor 2/4 (Ccr2/Ccr4), C-X3-C motif chemokine receptor 1 (Cx3cr1), growth-associated protein 45 gamma (GADD45G), and catenin delta 2 (CTNND2), with therapeutic strategies centered on high-density lipoprotein-like drug (HDLDG) targeting indole metabolism and gut microbiota modulation; for Alzheimer’s disease (AD), the core mechanisms involve epigenetic dysregulation of histone acetylation (H3K27ac: Histone H3 lysine 27 acetylation; H3K9ac: Histone H3 lysine 9 acetylation) mediated by CREB-binding protein/p300 (CBP/p300), impaired neurogenesis, and gut microbiota alterations (*Pseudomonas, Desulfovibrio, Enterococcus, Turicibacter, Rumellococcus*), with biomarkers including phenylacetylglutamine (PAGln), aquaporin-4 receptor (AQP4R), zinc finger protein 587B (ZNF587B), and C-reactive protein (CRP), and therapeutic approaches encompassing sphingolipid modulation via serine palmitoyltransferase long chain base subunit 2 (SPTLC2) and ceramide synthase 6A (CERS6A), upregulation of ATP-binding cassette transporter A1 (ABCA1), inhibition of carnitine palmitoyltransferase 1A (CPT1A), modulation of lysophosphatidylcholine acyltransferase (LCAC)/short-chain acyl-CoA (SCAC) metabolism, and fecal microbiota transplantation (FMT); for Parkinson’s disease (PD), the pathological cascade includes *α*-synuclein (SNCA) aggregation regulated by Nova alternative splicing regulator (NOVA1), lysosomal/autophagic dysfunction involving dynamin 1-like (DNM1L) and heat shock protein family A member 14 (HSPA14), extracellular matrix (ECM) remodeling, and exosome (EXOS) cluster formation, with additional molecular targets such as tyrosine hydroxylase 1 (YTH1) and IQ motif and WD repeats 1 (IQWD1), the biomarker Wiskott-Aldrich syndrome protein family member 1 (WASF12), and therapeutic strategies targeting C-X-C motif chemokine ligand 12 (CXCL12), the complement system (C3/C5), and dopamine (DA) neuron repair; for bacterial and viral meningitis, the schematic depicts temporal immune cell dynamics across early and acute stages, including CD8 + T cells expressing granzyme A (Gzma), T helper (Th) cells, plasmacytoid dendritic cells (pDC), regulatory T cells (Treg), and exhausted cytotoxic T lymphocyte subsets (MEM-CTL: Memory cytotoxic T lymphocytes; Tex: Exhausted T cells; Tpex: Progenitor exhausted T cells; Exh-pre: Precursor exhausted T cells; Exh-prg: Programmed exhausted T cells), alongside key markers such as memory T cells (Tmem), exhausted T cells with killer cell lectin-like receptor (Texh-KLR), programmed death ligand 1 (PD-L1), programmed death receptor 1 (PD-1), granzyme B (GZMB), complement component 5a (C5a), and cerebrospinal fluid (CSF) infiltration, with therapeutic strategies focused on modulating inflammation via lymphoid and myeloid cells including monocytes expressing thrombospondin 1 (THBS1), interleukin-1 beta (IL-1B), interferon-alpha/beta receptor subunit 2 (IFAR2), tumor necrosis factor (TNF), and proinflammatory and profibrotic factor 6 (PAFP6), as well as neutrophils; for multiple sclerosis (MS), core features include demyelination, tissue repair, and immune dysregulation, with molecular targets such as kinesin family member 5B (KIF5B), solute carrier family 1 member 3 (SLC1A3), and very late antigen 4 (VLA-4), biomarkers including T follicular helper cells (Tfh) and T helper 17 cells (Th17), and therapeutic interventions targeting T helper 1 (Th1)/Th17 cytokines, sphingolipid modulation via semaphorin 4D (Sema4D), Tie receptor 1/2 (TieR1/TieR2), cannabinoid receptor 3 (CannR3), and fatty acid-binding protein 5 (FABP5), complement component C1q, and astrocytic aquaporin 4 (AQP4) regulation, alongside disease-modifying therapies for secondary progressive multiple sclerosis (SPMS) and relapsing–remitting multiple sclerosis (RRMS); for autism spectrum disorder (ASD), the key pathways involve cerebellar telencephalon dysfunction, Rho guanine nucleotide exchange factor 6 (ARHGEF6)-mediated Rho GTPase signaling, and gamma-aminobutyric acid type B receptor subunit 2 (GABABR2) dysregulation, with additional mechanisms involving contactin-associated protein-like 2 (CNTNAP2), Ras-related C3 botulinum toxin substrate 1 (RAC1), and v-Ral simian leukemia viral oncogene homolog GTPase activating protein (v-Ral-GAP), the tricarboxylic acid (TCA) cycle as a biomarker, and therapeutic strategies targeting thyroid hormone receptor alpha (THRA), succinate dehydrogenase complex subunit A (SDHCA), and glutamate receptor (GluR) to modulate neuronal synaptogenesis; for depression, the core mechanisms include mitogen-activated protein kinase (MAPK)-mediated immune response and cell differentiation, synaptic plasticity, and neuroinflammation, with molecular targets such as synaptic vesicle-associated protein (SNAP), Ras homolog gene family member A (RhoA), and tissue factor pathway inhibitor (TFPI), biomarkers including interleukin-1 receptor antagonist (IL-1RA), C-reactive protein (CRP), and C-C motif chemokine ligand 2 (CCL2), and therapeutic approaches centered on synaptic plasticity modulation and neuroinflammation targeting.

Importantly, these selected diseases exhibit highly complementary and representative pathological mechanisms, enabling us to systematically reveal the common regulatory principles underlying diverse CNS disorders. Ischemic stroke and CNS infectious diseases are characterized by acute ischemic injury, excessive innate immune response, and neuronal apoptosis. AD and PD are defined by progressive protein misfolding and aggregation, as well as selective regional neuronal degeneration. MS is typified by autoimmune-mediated myelin damage and impaired neural signal transduction. ASD and MDD are primarily associated with neurodevelopmental abnormalities, neural circuit dysfunction, and neurotransmitter imbalance. By integrating analyses of these diverse disease models, we can systematically dissect the common regulatory pathways involved in CNS pathogenesis—including stress response, inflammatory signaling, autophagy dysregulation, RNA modification, and stress granule formation—thereby significantly enhancing the generalizability of our review framework (Wolozin and Ivanov, 2019; Hashmi et al., 2024). All these disease categories closely interfere with the normal physiological structure and functional homeostasis of the CNS, necessitating in-depth mechanistic exploration and precise therapeutic development with the support of single-Cell multi-omics technologies (Figure 3) (Table 2).

**Table 2:** Studies using single-cell multi-omics for the diagnosis and treatment of CNS disease.

Disease	Sample	Technique	Conclusion	Reference
IS	C57BL/6 mouse ttMCAO model	scRNA-seq	Top2A, Stmn1, Mki67, and Cdk1 in MG5 microglial cell subtypes and Cxcr2, S100a8, Il1b, and Mmp9 in MG6 cells can be considered as potential therapeutic targets.	Li et al. (2022)
IS	MCAO mouse and Sham groups	scRNA-seq	Microglial P2ry12, astrocytic GFAP, macrophage H2-Aa/H2-Ab1/Cd74, endothelial Spp1/Cxcl2/Ccl4/Cd14 can serve as unique biomarkers for tracking IS progression.	Zheng et al. (2022)
IS	C57BL/6 J male mouse tMCAO model	Transcriptomics, proteomics	GADD45G and CTNND2 are promising blood biomarkers for the prognosis and diagnosis of IS.	Simats et al. (2020)
IS	C57BL/6 J male mouse IS model	Metabolomics, transcriptomics	HQLDG protects mice from IS injury by modulating the gut microbiota-tryptophan metabolism-Th17/IL-17 signaling pathway.	Wu C. et al. (2024), Wu H. et al. (2024), Wu T. et al. (2024)
IS	C57BL/6 J male mouse sham and I/R groups	Proteomics, transcriptomics	Anxa2 may serve as a potential therapeutic target.	Tian et al. (2024)
AD	Postmortem human brain samples	Transcriptomics, proteomics, whole genome analysis	H3K27ac and H3K9ac are epigenetic drivers of AD, and they trigger disease pathways through transcriptional dysregulation and chromatin gene feedback loops.	Nativio et al. (2020)
AD	Human plasma and serum samples are divided into AD group and CN group.	Proteomics, metabolomics.	The proteins AQR, ZNF587B, CRP, and the metabolite PAGIn were identified as useful diagnostic indicators.	Yang J. et al. (2024), Yang K. et al. (2024)
AD	APPswe/PS1dE9 (APP/PS1) mice and their wild-type littermates are orally administered fingolimod at 1 mg/kg.	Metabolite-wide genomics, plasma metabolomics, lipidomics	The SM(d43:1)/SM(d34:1) ratio is a strong intermediate trait of sphingolipid dysregulation in AD. S1P metabolic regulators can serve as potential therapeutic candidates.	Baloni, et al. (2022)
AD	Samples are obtained from the first phase of the ADNI and its subsequent extensions.	Metabolomics, genetics, transcriptomics, proteomics	Adiponectin, CPT1A, and ABCA1 could serve as new biomarkers and therapeutic targets.	Horgusluoglu, et al. (2022)
AD	APP/PS1 transgenic mouse AD model and wild-type C57BL/6 J mice of the same litter	Spatial metabolomics, transcriptomics, 16S rRNA Sequencing	The abundance of gut microbiota in AD mice was significantly altered. FMT intervention reduced the levels of 1-stearoyl-2-hydroxy-sn-glycero-3-phosphocholine and PC (16:0/16:0) in the serum of APP/PS1 mice, while the level of phosphatidylcholine remained unchanged.	Qian X. et al. (2023), Qian Z. et al. (2023)
AD	381 ST samples across 16 Alzheimer’s disease-related studies and 1,053 sc/snRNA-seq samples from 85 studies	scRNA-seq, snRNA-seq, spatial transcriptomics	The transcriptomic changes in early-affected regions of AD may be more significant than those in late-affected areas. Additionally, neurogenesis pathway activity in female microglial cells was significantly upregulated, while in males, it was downregulated.	Wang et al. (2024)
PD	Pesticide-induced PD mouse model	scRNA-seq	Nova1 can act as a key regulatory factor in PD.	Khan et al. (2022)
PD	Collect peripheral blood samples from PD patients and healthy controls	scRNA-seq	NK cell-specific XCL2 could serve as a potential therapeutic target for PD.	Xiong et al. (2024)
PD	PPMI data includes the whole blood transcriptome data of PD patients as well as gene and clinical data from PD patients and the control group	Blood RNA-Seq, intron RNA transcriptomics	The changes in intron transcription occur synchronously with the clinical manifestations of PD, and WDFY3 can serve as a biomarker for PD and its progression.	Koks et al. (2022)
PD	Cell nuclei were extracted from the temporal cortex tissue of 12 PD patients and 12 control subjects.	Epigenomics, transcriptomics, snRNA-seq, snATAC-seq	Exc5 cluster is the only neuronal subtype showing upregulation of SNCA, and its specific tissue and synaptic structures are associated with disproportionate transcriptional dysregulation in PD.	Shwab et al. (2024)
PD	Data from single-cell transcriptomics, spatial transcriptomics, and proteomics from 17 high-quality studies were collected.	scRNA-seq, proteomics, transcriptomics.	It revealed the diversity of astrocytes and disease-specific transcriptional modules, with the expression of the M2 ECM module of astrocytes being elevated in PD.	Qian X. et al. (2023), Qian Z. et al. (2023)
PD	Midbrain samples from healthy controls and PD patients	epigenomics, snRNA-seq, snATAC-seq	With age, ODCs exhibit unique disease-related transcriptional profiles, which indirectly influences disease occurrence by affecting chromatin structure.	Adams et al. (2024)
VM	C57BL/6 mouse LCMV model	scRNA-seq, scATAC-seq	The stress response gene Btg1 as a novel regulator of Tex cells. Btg1 can mediate the transition of CD8 + T cell populations from Exh-Pre to Exh-Prog.	Giles et al. (2022)
VM	C57BL/6 mouse LCMV model	scRNA-seq, scTCR-seq, scBCR-seq	In chronic viral infections, a stronger NK cell response and Gzma high-expression effector CD8 + T cells were significantly induced at early stages, while acute infections notably induced more robust plasma cells and showed a decrease in but abnormal activation of pDCs.	Jin et al. (2024)
BM	Cultivate Group B Streptococcus (GBS; Agalactiae Streptococcus) with induced IMR90/C4 (WiCell) iPSC-derived hCMEC/D3 cells.	scRNA-seq	It was determined that the virulence factor for infection in BM is codY, which is a virulence factor of GBS. CodY is downregulated during the interaction between GBS and iBEC.	Vollmuth et al. (2024)
BM	A zebrafish model with fluorescently labeled *Streptococcus pneumoniae* D39VΔcomCDE mutant strain and its complementary strain.	scRNA-seq	PRR pathway was the key pathway of the pathogenesis of pneumococcal infection. Furthermore, necroptosis was identified as being crucial for host survival. However, the use of the drug GSK′872 could inhibit the necroptosis pathway.	Jim et al. (2022)
BM	Cerebrospinal fluid samples from pediatric bacterial meningitis patients	scRNA-seq	The macrophage-specific gene expression is higher in their own CSF cells, while platelet-specific gene expression is lower.	Xiao et al. (2022)
MS	C57BL/6 J and NOD/ShiLtJ(NOD mice)	RABID-seq	Microglia–astrocyte interactions mediated by Sema4D–PlexinB1, Sema4D–PlexinB2, and Ephrin-B3–EphB3 represent candidate therapeutic targets for intervention in MS.	Clark et al. (2021)
MS	Brain tissue blocks from 5 MS patients and 3 age- and sex-matched non-lesional, non-demented control individuals	snRNA-seq	PRLs–associated changes in MRI parameters at lesion edges may serve as potential biomarkers for assessing therapeutic effects in MS. Inhibition of C1q represents a promising therapeutic approach for treating MS.	Absinta et al. (2021)
MS	Human brain tissues from 10 individuals with PMS and 5 non-neurological disease controls	snRNA-seq, ATAC-seq, spatial transcriptomics	MS-specific oligodendrocyte gene signatures are associated with iron uptake and oxidative stress. Downregulation of SLC7A11 and VIM exacerbates their vulnerability to damage and impairs repair.	Elkjaer et al. (2024)
MS	Collect blood samples from 39 recruited MS patients and 40 recruited controls.	Proteomics, targeted metabolomics	TIMP-2 levels in SPMS patients are significantly lower than in RRMS patients and may serve as a biomarker to distinguish the two. Additionally, hippuric acid is proposed as a potential biomarker.	Zhou et al. (2024)
MS	Publicly available datasets	scRNA-seq, spatial transcriptomics, spatial proteomics	Ferroptosis can serve as a promising therapeutic target, as inhibiting ferroptosis can slow lesion progression. Ferroptosis-related genes in peripheral blood hold potential as diagnostic and prognostic biomarkers.	Wu C. et al. (2024), Wu H. et al. (2024), Wu T. et al. (2024)
MS	C57Bl/6 J mice EAE model	scFIND-seq, bulk FIND-seq	NR3C2 plays a role in limiting the reactive response of astrocytes that promote disease progression and may serve as a candidate therapeutic target.	Clark et al. (2023)
ASD	Stem cells and cerebral organoids were cultured.	scRNA-seq	In dorsal forebrain cells, progenitors differentiating into inhibitory neurons and oligodendrocytes—such as v-RGCs and INPs—are highly enriched. Disruption of the BAF complex components causes an accumulation of v-RGCs.	Li et al. (2023)
ASD	197 children with ASD and 299 healthy controls	Genomics, transcriptomics, proteomics	IGF1R is regulated by THRA and SCN2A and is associated with synaptic function. Variants primarily cluster in the Furin-like repeats domain, fibronectin type III domain, and tyrosine kinase catalytic domain, representing a potential pathogenic mechanism and therapeutic target for ASD.	Yang J. et al. (2024), Yang K. et al. (2024)
ASD	Fecal samples were collected from ASD patients. Male C57BL/6 J mice were administered a daily oral gavage of wild-type gut commensal *E. coli* at a dose of 10^8^, and fecal samples were collected accordingly.	Targeted metabolomics, metagenomics, 16S rRNA sequencing	An increased ratio of GABA to Glu serves as a diagnostic marker for autism. Imbalanced GABA metabolism is a prominent feature of the gut microbiota in children with mild ASD, and GABA may function as a key link between the gut microbiota and brain function.	Wang et al. (2025)
MDD	Public datasets.	scRNA-seq	YAP1 holds potential as a therapeutic target for depression.	Ma et al. (2024)
MDD	All patients completed questionnaires assessed using the BDI-II and the MADRS. Blood samples were collected.	scRNA-seq, proteomics	The high immune-related depression symptom cluster is characterized by increased BMI, more severe depression. The mild depression symptom cluster shows lower depression severity with no significant elevation in immune markers. The low immune-related depression symptom cluster features high depression severity without elevated immune markers.	Hagenberg et al. (2025)
MDD	Patients first underwent 12 weeks of acute escitalopram antidepressant treatment followed by 12 months of maintenance therapy. Peripheral blood samples were collected during the treatment process.	scRNA-seq, ATAC-seq	A reduction in CD4^+^ naive T cells can serve as a predictive marker for poor response to antidepressant treatment. Activation of CD4^+^ naïve T cells mediated by the ETS-MAPK pathway characterizes patients with major depression who show a positive response to antidepressant therapy.	Sun et al. (2024)

IS, Ischemic Stroke; AD, Alzheimer’s Disease; PD, Parkinson’s Disease; VM, Viral Meningitis; BM, Bacterial Meningitis; MS, Multiple Sclerosis; ASD, Autism Spectrum Disorder; MDD, Major Depressive Disorder.

### 3.1 Cerebrovascular diseases

Cerebrovascular disease (CeVD) encompasses a heterogeneous group of vascular disorders affecting the cerebral circulation, mainly including cerebral artery stenosis and intracranial aneurysms. Cerebral artery stenosis is primarily induced by atherosclerosis, which can further trigger vascular embolism and cerebral ischemia; intracranial aneurysms, by contrast, are prone to rupture and subsequently cause intracranial hemorrhage. Both ischemic and hemorrhagic events ultimately lead to the occurrence of stroke. CeVD markedly impairs patients’ quality of life and frequently results in long-term neurological disability and even mortality, ranking it as the second leading cause of death globally (Andjelkovic et al., 2022). Epidemiological data indicate that approximately 85% of CeVD-related strokes are ischemic, whereas the remaining 15% are hemorrhagic (Kumar et al., 2023). Given the high prevalence and clinical representativeness of IS among CeVD subtypes, the following section takes IS as a typical representative to elaborate the applications of single-cell multi-omics technologies in exploring the pathogenesis and therapeutic targets of IS.

Li et al. (2022) used scRNA-seq experiments to discover that in microglial subtypes, the genes Top2A, Stmn1, Mki67, and Cdk1 of the MG5 cells were significantly upregulated in the elderly brain following IS. The MG6 cells showed upregulated expression of Cxcr2, S100a8, Il1b, and Mmp9, exhibiting a unique “neutrophil-like” phenotype. This novel subtype may represent an IS-specific state of microglia in the aged brain post-IS. However, this study only explored the IS-specific states of microglial subtypes and lacked investigation into the states of other cell subtypes after IS. Furthermore, Guo et al. (2021) found through scRNA-seq that microglia are among the first glial cells to respond to cerebral blood flow interruption and can differentiate into M1 and M2 phenotypes. During the acute phase, M1 microglia are enriched with inflammation-related genes and hypoxia pathways, while M2 microglia participate in repair processes, neurogenesis, axonal remodeling, and angiogenesis. Astrocytes, as one of the pathophysiological hallmarks of IS, play roles in blood–brain barrier formation and neuronal energy metabolism. When gap junctions are impaired, astrocytes release inflammatory mediators. High expression of Cyr61 promotes astrocyte survival in IS. Macrophages promote inflammatory processes; their infiltration and polarization can be detected in the early stage of IS. Overexpression of Mmp9 and Mmp8 leads to physical disruption of the blood–brain barrier (BBB), thereby affecting brain tissue. Oligodendrocytes can participate in the formation of the BBB and neuronal energy metabolism. Sgk3 is highly expressed in oligodendrocytes and can regulate cell survival and inflammatory responses. Endothelial cells, as a component of the BBB, are involved in vascular remodeling and extracellular communication and are considered early therapeutic targets. However, this study lacked sufficient investigation into potential intercellular communications in the IS brain. Zheng et al. (2022) applied scRNA-seq to construct cellular atlases in a mouse model of cerebral artery occlusion. Their study showed that the molecular basis of IS mainly involves neuronal injury, including neuronal necrosis and apoptosis, ischemic damage, therapeutic responses, and infiltration of inflammatory cells. Neuronal injury is caused by neuronal loss, oxidative stress, and immune responses. The interaction between microglia in the CNS, resident brain cells, and infiltrating immune cells may dominate neuroinflammation in IS, which triggers the upregulation of key cell type-specific genes. Thus, cell type-specific differentially expressed genes (DEGs) affected by ischemic injury can serve as unique biomarkers to track IS progression, including P2ry12 in microglia, GFAP in astrocytes, H2-Aa, H2-Ab1, and Cd74 in macrophages, and Spp1, Cxcl2, Ccl4, and Cd14 in endothelial cells. It was also found that communication between fibroblasts and other cells decreased in IS, whereas microglia- and meningeal macrophage-dominated intercellular interactions significantly increased, especially predicted interactions between microglia and other immune cells, astrocytes, pericytes, and oligodendrocytes were more pronounced. In summary, scRNA-seq reveals precise transcriptional changes during neuroinflammation at the single-cell level, opening new avenues for exploring IS disease mechanisms and identifying potential novel biomarkers and therapeutic targets based on cell subtype-specific molecules.

However, the above content only describes genes individually at the single-cell level. While it provides a reliable understanding of the main mechanisms of IS at different molecular levels, it still lacks elaboration on the interactions and connections between genes, proteins, and metabolism. The application of multi-omics can make up for this gap. Simats et al. (2020) established a transient cerebral ischemia model found that the expression of the GADD45G gene in the brain increased significantly in the early stage after ischemia; this was because the rapid increase in mRNA levels translated into higher GADD45G protein content. In ischemic brain tissue, the expression of the CTNND2 gene and its protein content decreased abruptly, while the level of CTNND2 in the blood increased. Since CTNND2 expression is highly specific to the brain, it was hypothesized that the increase in blood CTNND2 content might be a direct result of release from ischemic brain tissue. Based on the above analysis, it was confirmed that GADD45G and CTNND2 are promising blood biomarkers for IS prognosis and diagnosis, respectively. Wu C. et al. (2024) established an IS mouse model using the middle cerebral artery occlusion method and conducted experiments. Transcriptomic analysis results showed that HQLDG significantly reduced infarct volume and neurological scores. Metabolomic analysis results revealed that the HQLDG treatment group could regulate tryptophan (Trp) metabolic pathways, such as the contents of indoleacetaldehyde, indolelactic acid, and L-Trp in serum.

Furthermore, tryptophan-targeted metabolomics further confirmed these results, demonstrating that HQLDG could significantly regulate metabolites in the indole pathway of tryptophan metabolism. Based on the above analysis, it was confirmed that HQLDG protects mice from IS damage by regulating the gut microbiota-tryptophan metabolism-Th17/IL-17 signaling axis. Tian et al. (2024) conducted experiments by establishing a cerebral ischemia–reperfusion model and identified that Anxa2 as a key molecule regulating the immunoinflammatory response of microglia. Anxa2 is essential for microglial activation and the production of proinflammatory factors by promoting the nuclear translocation of the p65 subunit via the nuclear factor kappa-light-chain-enhancer of activated B cells (NF-κB) signaling pathway. Deletion of Anxa2 expression impairs the gene expression profile associated with microglial inflammation, and Anxa2 deletion reduces neuronal death in the oxygen–glucose deprivation and reoxygenation (OGD/R) response in a non-cell-autonomous manner. This study revealed the important role of microglial Anxa2 in regulating inflammatory responses, suggesting that ANXA2 may be a potential therapeutic target for IS. In summary, the combined application of single-cell sequencing and multi-omics, from gene to protein to metabolite levels, demonstrates the precise transcriptional changes of genes and proteins in IS cells, providing new insights into the pathogenesis of IS, blood biomarkers, potential novel biomarkers, and therapeutic targets.

### 3.2 Neurodegenerative diseases

Neurodegenerative diseases are a heterogeneous and complex group of disorders characterized by the gradual loss of structure and function of neurons, ultimately leading to neuronal death (Agnello and Ciaccio, 2022). Approximately 50 million people worldwide suffer from AD, and about 10 million have PD (Zwirner et al., 2022). However, most patients with neurodegenerative diseases currently have no cure, and treatment mainly focuses on symptom relief and improving quality of life. Next, we will specifically explore the applications of single-cell sequencing and multi-omics technologies in the pathogenesis, diagnostic methods, and treatment of AD and PD—the two most common neurodegenerative diseases (Ruz et al., 2020).

#### 3.2.1 Alzheimer’s disease

AD is the most common type of dementia, accounting for 60 to 80% of all cases. Nativio et al. (2020) performed transcriptomic analyses on young, elderly, and AD patients, revealing upregulation of transcription- and chromatin-associated genes such as CBP/P300 in AD, which mediate H3K27ac and H3K9ac modifications. Proteomic and genomic analyses demonstrated a significant increase of H3K27ac and H3K9ac in AD, correlating with functional categories of transcription and chromatin genes. At the epigenetic level, the disease-specific increase of H3K9ac on CREBBP suggests a potential positive feedback loop that promotes sustained CBP expression in AD, thereby maintaining elevated levels of H3K27ac. Integrative analyses of transcriptomics, proteomics, and genomics identify H3K27ac and H3K9ac as possible epigenetic drivers of AD, which contribute to disease pathways through transcriptional dysregulation and chromatin gene feedback loops, providing mechanistic insights into AD progression. Yang J. et al. (2024) applied high-resolution mass spectrometry to plasma and serum samples to investigate senescence-associated secretory phenotype (SASP) related to AD. Proteomic analysis showed that five plasma proteins were upregulated in AD patients. Metabolomic analysis revealed elevated levels of phenylacetylglutamine (PAGln) in AD patient serum. Mass spectrometry-based profiling enabled simultaneous measurement of multiple biomarkers in the plasma proteome and metabolome of AD patients, significantly enhancing biomarker discovery efficiency. The employed machine learning algorithm, k-nearest neighbors (KNN), demonstrated specificity and accuracy within a comprehensive diagnostic framework. Based on the above analyses, proteins AQR, ZNF587B, CRP, and the metabolite PAGln have been identified as useful diagnostic biomarkers for AD.

Baloni et al. (2022) constructed an amyloid protein-producing APP/PS1 mouse model and performed Metabolite genome-wide association analysis. The results showed that SPTLC3, CERS2, and CERS4 are involved in the synthesis of sphingomyelin, while SPHK2, SGPP1, and SGPL1 play roles in the synthesis and degradation of sphingosine-1-phosphate (S1P). As a precursor to S1P, changes in sphingomyelin species such as SM(d33:0), SM(d34:1), and SM(d38:2) directly influence S1P production and degradation. S1P, an intermediate metabolite of sphingolipid metabolism, plays a crucial role in AD pathology. Plasma metabolomics and lipidomics identified the ratio of SM(d43:1) to SM(d34:1) as a strong intermediate phenotype of sphingolipid dysregulation in AD. Metabolite genome-wide association analysis (mGWAS) further validated sphingosine-1-phosphate (S1P) metabolites as potential therapeutic targets for AD and demonstrated that treatment with fingolimod alleviated synaptic plasticity deficits and cognitive impairment in mice. Taken together, these multi-omics analyses highlight potential targets within the SM pathway and suggest that modulation of S1P metabolism may serve as a candidate therapeutic strategy for AD.

Horgusluoglu et al. (2022) performed metabolite co-expression network analysis on blood metabolomic data from the Alzheimer’s Disease Neuroimaging Initiative (ADNI). They found that short-chain acylcarnitines/amino acids and medium-to-long-chain acylcarnitines were most closely associated with AD clinical outcomes, while ABCA1 and CPT1A played roles in regulating acylcarnitines and amino acids. A constructed multi-scale embedded gene co-expression network analysis showed that subnetworks centered on CPT1A and ABCA1 were associated with the neuronal system and immune response, respectively. Testing of bulk tissue metabolomic data from ADNI using Slingshot revealed that the expression levels of short-chain acylcarnitine and amine modules decreased over time, while those of medium-to-long-chain acylcarnitine modules increased over time. Comprehensive analysis showed that elevated ABCA1 mRNA and adiponectin protein levels corresponded to reduced short-chain acylcarnitine and amino acid levels in AD patients. Adiponectin, CPT1A, and ABCA1 were identified as novel biomarkers and therapeutic targets; furthermore, increased circulating adiponectin levels and enhanced ABCA1 expression were found to be compensatory effects against neurodegeneration. Qian X. et al. (2023) analyzed 9-month-old APP/PS1 and wild-type (WT) mouse models using spatial metabolomics and transcriptomics. They found that in APP/PS1 mice, the abundance of gut microbiota was significantly altered. Using 16S rRNA sequencing, differential gene expression in the brains of APP/PS1 and WT mice was analyzed, showing pronounced proliferation and activation of astrocytes and microglia in APP/PS1 mice, accompanied by changes in immune pathways. Fecal microbiota transplantation (FMT) interventions were employed to validate multi-omics findings, demonstrating that FMT reduced serum levels of 1-stearoyl-2-hydroxy-sn-glycero-3-phosphocholine and PC (16:0/16:0) in APP/PS1 mice, while phosphatidylcholine levels remained unchanged, indicating partial modulation of glycerophospholipid metabolism by FMT. Collectively, these analyses reveal a potential link between gut microbiota, host glycerophospholipid metabolism, and neuroinflammation in APP/PS1 mice, advancing the understanding of the relationship between gut microbiota and AD.

While the above multi-omics-based studies have provided clear insights into the mechanisms, diagnosis, and biomarkers of AD, limitations remain. There is a disconnect between research models and clinical translation: many studies rely on single transgenic mouse models such as APP/PS1, whose pathological features differ from the complexity of human AD. Additionally, studies on clinical samples (e.g., plasma, cerebrospinal fluid) mostly use small samples or single-center designs, lacking large-scale, multi-center clinical validation. This casts doubt on the clinical applicability and reliability of the discovered biomarkers and therapeutic targets. The combination of single-cell sequencing and multi-omics technologies will bring new ideas for exploring personalized AD treatment strategies. Wang et al. (2024) performed transcriptomic analysis of DEGs between AD and control cells revealed that early-affected brain regions exhibited more pronounced transcriptomic alterations than late-affected regions such as the prefrontal cortex. Moreover, comparative analysis of gene expression between males and females uncovered a significant upregulation of neurogenesis pathways in female microglia under AD conditions, contrasting with a downregulation in males. This study, integrating scRNA-seq, snRNA-seq, and spatial transcriptomics, elucidated biological pathways and regulatory networks associated with AD progression, highlighting the influence of sex on the cellular transcriptomic landscape. These findings provide new insights into the complex mechanisms of AD and pave the way for the development of more personalized, sex-specific therapeutic strategies. In summary, the combined application of single-cell sequencing and multi-omics technologies, encompassing gene expression, proteomics, plasma metabolomics, and lipidomics, has revealed precise transcriptional and proteomic alterations in AD patient cells, offering new perspectives on AD pathogenesis, diagnostic methods, biomarker discovery, therapeutic targets, and personalized sex-specific treatment approaches.

#### 3.2.2 Parkinson’s disease

PD is the second most common age-related neurodegenerative disorder after Alzheimer’s disease, pathologically characterized by progressive loss of dopaminergic neurons in the substantia nigra pars compacta of the midbrain and the formation of intracellular eosinophilic inclusion bodies termed Lewy bodies. The major component of Lewy bodies is abnormally aggregated and phosphorylated *α*-synuclein (α-Syn), which is regarded as the core pathological biomarker of PD. The continuous accumulation and spread of misfolded α-Syn further trigger neuroinflammation, synaptic dysfunction, and progressive neurodegeneration, ultimately leading to typical motor symptoms including bradykinesia, resting tremor, rigidity, and postural instability, as well as a variety of non-motor symptoms such as sleep disturbance, cognitive decline, and emotional disorders. Khan et al. (2022) found that in a PD mouse model treated with a combined administration of two doses [maneb and paraquat (MNPQ)], the mice exhibited loss of tyrosine hydroxylase-positive neurons in the substantia nigra pars compacta (SNpc), increased α-synuclein aggregates, and motor abnormalities resembling those observed in human PD. scRNA-seq of the SNpc revealed 14 genes with significantly differential expression across four or more cell types, indicating that both cell-specific and non–cell-specific pathways contribute to pesticide-induced PD pathogenesis. Network analysis of all DEGs using InnateDB identified Nova1, an important regulator of alternative splicing, as being significantly upregulated in SNpc neurons following MNPQ treatment compared to controls, implicating Nova1 as a critical regulatory factor in PD. Xiong et al. (2024) employed scRNA-seq on peripheral blood mononuclear cells to explore the relationship between PD severity and peripheral immune responses, the study found a significant reduction of natural killer (NK) cells in blood samples as PD advanced, alongside evidence of NK cell infiltration into the motor cortex, confirming a close interaction between peripheral immune responses and the CNS. Moreover, NK cells were enriched in mitochondrial-related processes, which are linked to dopaminergic neuron dysfunction. scRNA-seq data further demonstrated that expression of XCL2 markedly increased in NK cell subpopulations C2 and C5 with worsening PD severity, suggesting that XCL2 could serve as a valuable target for future PD therapeutic strategies. It is evident that high-throughput scRNA-seq has become a primary method for characterizing cellular heterogeneity in neurological diseases.

However, it has limitations in dynamic and spatial dimension research: existing studies only capture static cellular and gene expression characteristics at specific stages of PD, and fail to analyze the temporal evolution of cell subtypes and molecular expression during disease progression through dynamic tracking technologies. Meanwhile, the lack of spatial transcriptomic analysis makes it impossible to clarify the spatial localization of differentially expressed genes in SNpc and the intercellular interaction relationships, which hinders the complete reconstruction of the spatial characteristics of the pathological microenvironment in the PD brain.

Due to the difficulty in obtaining human brain specimens, most studies have been unable to deeply elucidate the specific disease mechanisms of PD. The integration of single-cell sequencing with multi-omics technologies offers novel avenues for exploring PD pathogenesis. The prospect of applying multi-omics to PD research was proposed as early as 2018. Trevisan et al. (2024) emphasized that a key goal in PD treatment is identifying suitable biomarkers and suggested expanding clinical sample collection beyond plasma/serum to include urine, saliva, sweat, or intraocular fluids, aiming to improve early-stage data acquisition. Koks et al. (2022) analyzed intronic expression in the Parkinson’s Progression Markers Initiative (PPMI) cohort and found that compared to diagnosis, 4,873 intronic transcripts exhibited differential expression in PD patients 3 years later—for example, WDFY3—indicating that changes in intronic transcription occur synchronously with PD clinical manifestations and may serve as biomarkers for PD and its progression. Based on the analysis of blood RNA-Seq and intronic transcriptomics, it was found that the genes DNAJC19 and TIMM14 encode inner mitochondrial membrane translocases directly involved in neurodegenerative disease mechanisms. The TIMM gene products interact with outer mitochondrial membrane translocases, forming transport pathways for nuclear-encoded precursor proteins. Shwab et al. (2024) employed parallel single-nucleus snRNA-seq and snATAC-seq technologies to successfully identify a glutamatergic excitatory neuron subpopulation, termed the Exc5 cluster, which exhibited numerous DEGs including YY1, SP3, and KLF16, and was the sole neuronal subtype showing upregulation of *α*-synuclein (SNCA). Overexpression of SNCA is closely associated with PD pathogenesis. Furthermore, Exc5-specific tissue and synaptic architecture were linked to disproportionate transcriptional dysregulation in PD. By combining multi-Omics analyses, the researchers gained novel insights into cell subtype-specific gene expression dysregulation mechanisms in PD. YY1 functions as both a transcriptional activator and repressor and plays a critical role in neuronal development and function; SP3 is involved in various developmental processes and inflammatory NF-κB signaling; KLF16 inhibits synaptic growth and modulates dopaminergic synaptic transmission in the striatum.

Qian Z. et al. (2023) took astrocytes as the entry point and, through large-scale integration of scRNA-seq data, identified seven shared expression modules from astrocytes. At the transcriptomic level, these modules showed significant heterogeneity across different diseases. These modules were validated at the protein level using mass spectrometry datasets, and ligand-receptor signaling pathways were further explored. Multi-omics analyses combining scRNA-seq, proteomics, and transcriptomics revealed upregulation of the M2 extracellular matrix (ECM) module in astrocytes within PD, providing new insights into PD pathogenesis. Adams et al. (2024) analyzed snRNA-seq and snATAC-seq data from the substantia nigra region of young control groups, elderly control groups, and PD patients. They identified cell type-specific changes in gene expression among these groups and found that carnosine synthase 1—encoded by the CARNS1 gene and possessing antioxidant and neuroprotective effects—decreases with increasing age and PD progression. Integrating multi-Omics data, the study elucidated how aging elevates PD risk: with increasing age, oligodendrocytes (ODCs)—a principal midbrain cell type—exhibit unique disease-associated transcriptional features, and changes in gene activation or repression affect cellular function. Moreover, the presence of increased shared ATAC peaks among different cell types enhances the SNP heritability of PD by indirectly influencing chromatin structure and thus disease occurrence. In summary, the integration of single-cell sequencing with multi-omics at the gene, protein, metabolite, and transcriptomic levels has revealed precise transcriptional alterations in PD patient cells, providing new perspectives for understanding PD pathogenesis, biomarker discovery, and risk assessment in the elderly population.

### 3.3 Central nervous system infectious diseases

CNS infectious diseases refer to acute or chronic inflammatory conditions caused by various pathogens invading the CNS, including the brain parenchyma, meninges, and vasculature. Common types of infections include viral, bacterial, fungal, and parasitic infections. A study conducted in England between 2011 and 2014 reported an incidence rate of VM of 2.73 cases per 100,000 population, with non-pneumonia viruses accounting for the majority of cases. Additionally, a study in China found that VM caused by Enterovirus 71 (EV-71) accounted for approximately 55.2% of nervous system diseases (Kohil et al., 2021). Research spanning 1935 to 2019 across 108 countries revealed a mortality rate of 18% for BM, with Listeria meningitis exhibiting the highest mortality at 27%, followed by *Streptococcus pneumoniae* at 24%, *Neisseria meningitidis* at 9%, and *Haemophilus influenzae* at 11% (van Ettekoven et al., 2024). These data highlight the significant threat that viral and BM pose to human health. The following section discusses the application of single-cell multi-omics technologies in elucidating cellular alterations and therapeutic strategies in VM and BM.

#### 3.3.1 Viral meningitis

Giles et al. (2022) used a lymphocytic choriomeningitis virus model and analyzed the heterogeneity and developmental trajectories of effector T cell (Teff), memory T cell (Tmem), and exhausted T cell (Tex) cells differentiated from CD8 + T cells through longitudinal scRNA-seq and scATAC-seq. The study found that the number of clusters defined by scATAC-seq was smaller than that by scRNA-seq, indicating that cells may be epigenetically similar but still exhibit diversity in gene expression patterns. Secondly, single-cell analysis revealed new subsets of Teff, Tmem, and Tex cells, including an NK receptor-expressing Tex subset and an early Tmem subset characterized by cytotoxic potential. Combined with epigenetic analysis, the researchers identified epigenetically distinct T cell factor 1 (TCF-1) + CD8 + T cell populations in chronic infections and acutely resolving infections; among these, TCF-1-positive populations shared some transcriptional characteristics. However, these subsets had unique and accessible chromatin landscapes, which further evolved over time during the development of Tmem and Tex cells. Finally, the experiment identified the stress response gene Btg1 as a new regulator of Tex cells, which can mediate the transition of CD8 + T cell populations from Exh-Pre to Exh-Prog. This is of key significance for understanding the immune response to treatment and identifying clinical biomarkers.

Jin et al. (2024) used a mouse lymphocytic choriomeningitis virus model and explored the immune response landscape of acute and chronic VM via high-throughput scRNA-seq. The analysis showed that compared with acute viral infection, chronic viral infection significantly induced stronger NK cell responses in the early stage and stronger plasma cell responses in the acute stage. In addition, chronic viral infection led to a reduced number of plasmacytoid dendritic cells (pDCs) in the acute stage, which were abnormally activated. Through single-cell B cell receptor sequencing (scBCR-seq), it was found that plasma cells increased significantly in chronic infections, but there were differences in the usage of B cell receptors. Single-cell T cell receptor sequencing (scTCR-seq) revealed that in the early stage of chronic infection, effector-like CD8 + T cells with high Gzma expression were significantly induced; these cells showed a time-dependent reversal of gene expression patterns throughout the viral infection process, and the dominant TCR type they used differed from that in other stages. By integrating single-cell RNA-seq and single-cell antibody sequencing, the study found that chronic infection induced a stronger CD4 + T cell response (including germinal center helper T cells and regulatory T cells) and disrupted the TCR diversity of CD8 + and CD4 + T cells. These findings revealed the longitudinal dynamics and heterogeneity of lymph node immune cells during acute and chronic viral infections.

In summary, at the single-cell level, single-cell sequencing has revealed precise transcriptional changes in the cells of VM patients, providing new insights into the changes of immune cells in VM. However, it has limitations in dynamic research: although the former study involved longitudinal analysis, it did not integrate spatial transcriptomics technology, making it impossible to clarify the spatial localization of differential cell subsets in tissues and the local interaction relationships between cells; while the latter study focused on the phase differences between acute and chronic infections, it failed to analyze the temporal patterns of cellular and molecular changes during the VM disease course. This makes it difficult to identify the key nodes of pathological progression and intervention windows, resulting in an incomplete understanding of the dynamic evolution mechanism of VM.

#### 3.3.2 Bacterial meningitis

BM typically occurs when bacteria penetrate the BBB or the meningeal–brain–cerebrospinal fluid barrier (mBCSFB), both of which are composed of highly specialized brain endothelial cells (BECs). Group B Streptococcus (GBS) is a major pathogen responsible for neonatal meningitis. Accordingly, Vollmuth et al. (2024) performed scRNA-seq analysis on the interaction between GBS and induced pluripotent stem cell-derived brain endothelial cells (iBECs) to identify virulence-associated genes. Among 2,068 annotated protein-coding genes in GBS, 430 transcripts showed significant expression changes following interaction with BECs, with most differentially expressed GBS transcripts being downregulated during iBEC infection. scRNA-seq analysis revealed downregulation of codY in GBS during interaction with iBECs. CodY mutants in three distinct GBS backgrounds demonstrated increased adhesion and invasiveness in two *in vitro* BEC models, as confirmed by quantitative polymerase chain reaction (qPCR). This study delineated virulence factors at the transcriptomic level but did not fully explore the pathways involved in BM pathogenesis.

Jim et al. (2022) used the whole-animal *in vivo* dual RNA-seq method and found that intercellular communication and intercellular heterogeneity of *Streptococcus pneumoniae* are involved in the pathogenesis of *Streptococcus pneumoniae* infection. Functional enrichment analysis pinpointed host pattern recognition receptor (PRR) pathways activated by pneumolysin, with necroptosis playing a crucial role in host survival. Pharmacological inhibition of necroptosis via GSK′872 increased mortality in pneumococcal meningitis, highlighting necroptosis’s protective function. These transcriptomic insights shed light on pneumolysin-specific host responses and identified key pathways involved in BM, yet emphasized the need for enhanced cellular-level diagnostic approaches. Cerebrospinal fluid (CSF) cytology is a critical parameter for BM diagnosis, and elucidating cellular heterogeneity within CSF can greatly enhance understanding of BM. Xiao et al. (2022) collected CSF samples from a large number of BM patients at different disease progression stages, and performed scRNA-seq as well as other bulk transcriptome sequencing. The study found that with the progression of the disease, the proportion of myeloid cells decreased while the proportion of lymphoid cells increased. Through scRNA-seq characterization, the study revealed that NEUS and MOS in BM patients are the main cell types in CSF. It also discovered a new subtype of neutrophils and a new subtype of monocytes; the changes in their quantities were positively correlated with the intensity of inflammatory responses in CSF, which indicates that the main neuroinflammatory response in BM patients is innate immunity. By analyzing the overall transcriptome profiles of autologous CSF cells and peripheral blood leukocytes in BM patients, the researchers found that compared with peripheral blood leukocytes, the expression of macrophage signature genes was higher and the expression of platelet signature genes was lower in the autologous CSF cells of BM patients. It is evident that this study uncovered the heterogeneity of CSF cells in BM at the single-cell level and provided new insights into the involvement of immune cells in CNS infections. In summary, the combined application of single-cell sequencing and multi-omics has revealed precise genetic and transcriptomic changes in the cells of BM patients from the genetic to the transcriptional level, offering new ideas for exploring the virulence factors, infection pathways, and cellular heterogeneity of BM.

### 3.4 Central nervous system demyelinating diseases

CNS demyelinating diseases are characterized primarily by the loss or damage of myelin sheaths surrounding central nerve fibers, encompassing hereditary, infectious, and autoimmune etiologies. Among these, MS is the most prevalent chronic autoimmune demyelinating disease of the CNS (Hauser and Cree, 2020). Clinically, MS is classified according to disease course into relapsing–remitting MS (RRMS), primary progressive MS (PPMS), and secondary progressive MS (SPMS)(Kuhlmann et al., 2023). The following summarizes applications of single-cell sequencing and multi-omics technologies in elucidating MS pathogenesis and therapeutic strategies.

Clark et al. (2021) developed rabies barcode interaction detection followed by sequencing (RABID-seq) by combining viral barcode tracing with scRNA-seq to study cell–cell interactions of astrocytes in mouse CNS *in vivo*. Their findings identified that Sema4D and Ephrin-B3 are expressed in microglia and regulate astrocyte responses via PlexinB1, PlexinB2, and EphB3 receptors, respectively. This implicates Sema4D-PlexinB1, Sema4D-PlexinB2, and Ephrin-B3–EphB3 signaling pathways as modulators of microglia-astrocyte crosstalk and potential therapeutic targets for MS intervention. Absinta et al. (2021) employed snRNA-seq to analyze human brain tissue, and found that the transcriptomic profiles of inflammatory microglia in MS (MIMS) overlap with microglial profiles observed in other neurodegenerative diseases, suggesting shared mechanisms between primary and secondary neurodegeneration. Utilizing an autoimmune encephalomyelitis mouse model with microglia-specific C1q knockout, the study identified complement component C1q as a key mediator of MIMS activation, highlighting C1q inhibition as a promising therapeutic strategy for MS. Collectively, these studies demonstrate that single-cell sequencing elucidates the microglia-astrocyte interactions and identifies candidate therapeutic targets at the single-cell level, advancing mechanistic understanding and treatment approaches for MS.

The integration of single-cell sequencing and multi-omics approaches offers innovative perspectives for elucidating the pathogenesis and therapeutic strategies of MS. Elkjaer et al. (2024) employed snRNA-seq, ATAC-seq, and spatial transcriptomics to identify that MS-specific oligodendrocyte gene signatures are predominantly regulated by the KLF/SP transcription factor family, with functional associations to iron uptake and oxidative stress pathways. These oligodendrocyte populations exhibited upregulation of pro-inflammatory molecules, while downregulation of SLC7A11 and VIM was implicated in enhanced vulnerability to injury and impaired remyelination capacity. Furthermore, a distinctive metabolic astrocyte phenotype was characterized within the core and rim regions of chronic active lesions, alongside the discovery of FGF13-FGFR3 co-expression between astrocytes and neurons, implicating this axis in lesion repair processes. Additionally, B cell co-expression network analyses revealed discrete B cell subsets with spatially and functionally divergent distributions across lesion types, advancing our understanding of the heterogeneity underlying progressive MS pathology. Zhou et al. (2024) used proteomic analyses to identify significant downregulation of neurotrophic factors and proteins related to tissue repair, including persephin, NT-3, BDNF, IGFBP-1, IGFBP-2, and EGF. Metabolomic profiling further highlighted marked decreases in anti-inflammatory mediators and sphingolipids, the latter potentially contributing to defective remyelination. Integrated multi-omics comparisons delineated distinct peripheral blood biomarker signatures among clinical subtypes; notably, clinical isolated syndrome (CIS) patients demonstrated elevated levels of TRAIL-R3 and other molecules relative to RRMS and SPMS, while SPMS patients exhibited significantly reduced TIMP-2 compared to RRMS, suggesting utility as a differential biomarker. Kynurenic acid levels inversely correlated with disease severity, underscoring its potential as a prognostic marker. These data provide critical insights for biomarker-driven stratification and pathophysiological interrogation of MS clinical phenotypes.

Wu T. et al. (2024) investigated ferroptosis involvement in MS pathophysiology via integrated snRNA-seq, spatial transcriptomics, and spatial proteomics. They identified peak ferroptosis scores at the rims of active white matter lesions, correlating with microglial activation and iron deposition patterns, implying that ferroptosis inhibition may retard lesion progression. Conversely, remyelinating lesion areas exhibited minimal ferroptosis activity. Mechanistically, ferroptosis contributed to demyelination through pathways including phagocytosis of iron-laden myelin debris, pro-inflammatory polarization, and lipid peroxidation, with ferroptotic cells enhancing T cell activation. Spatial analyses demonstrated a negative correlation between ferroptosis scores and neurodegeneration markers in cortical neurons, whereas positive correlations were observed in microglia. These findings reveal a dual role for ferroptosis in MS, bridging neuroimmune activation and neurodegeneration, highlighting it as a promising therapeutic target. Furthermore, expression of ferroptosis-related genes in peripheral blood and CSF showed positive correlation, and a diagnostic model based on 24 ferroptosis-associated genes exhibited robust performance in MS diagnosis and relapse prediction, supporting their potential as minimally invasive biomarkers. Clark et al. (2023) developed a novel nucleic acid-based cell interrogation technology (FIND-seq) to isolate rare cell populations defined by nucleic acid features. Combining CRISPR-Cas9 genetic perturbations in mouse models with scFIND-seq profiling in experimental autoimmune encephalomyelitis (EAE) and human MS, they revealed downregulation of NR3C2 signaling in Aqp4 + Edem1 + astrocytes. Astrocyte-specific knockdown of the nuclear receptor NR3C2 exacerbated EAE pathology, indicating that NR3C2 functions to restrain pro-inflammatory astrocyte responses and may represent a viable therapeutic target. Additionally, NCOR2-mediated recruitment of histone deacetylases to chromatin repressed XBP1-driven transcriptional programs, providing an epigenetic regulatory mechanism with potential for therapeutic exploitation in MS. In conclusion, the synergistic application of single-cell and multi-omics technologies across genomic, transcriptomic, proteomic, and metabolomic dimensions has elucidated intricate cellular crosstalk and precise molecular alterations in MS. These advances significantly deepen our understanding of MS pathogenesis, facilitate differentiation of clinical subtypes, and support the development of novel diagnostic, prognostic, and therapeutic strategies.

### 3.5 Psychiatric disorders

Psychiatric disorders refer to a range of conditions characterized by brain dysfunction caused by biological, genetic, behavioral, and other multifactorial influences, manifesting as disordered thinking, abnormal emotions, and atypical behaviors that severely impact an individual’s daily life and social functioning (Goldfarb et al., 2022). Common categories include neurodevelopmental disorders, anxiety and fear-related disorders, eating disorders, stress-related disorders, personality disorders, and mood disorders, among others (Solmi et al., 2022). A meta-analysis of 71 studies since 2012, encompassing 99 estimates, indicated a global median prevalence of ASD of 10 per 1,000 individuals (range: 1.09–436 per 10,000), with a median proportion of ASD cases accompanied by intellectual disability at 33.0%. Approximately 1 in every 100 children is diagnosed with ASD, with prevalence showing an increasing trend worldwide (Zeidan et al., 2022). Roddy Mitchell et al. (2023) analyzed 589 reports covering 616,708 women from 51 countries and found that 1 in 4 perinatal women experience depression. Undoubtedly, ASD and depression have become major threats to human mental and physical health. The following section summarizes the application of single-cell multi-omics technologies in elucidating cellular changes and therapeutic approaches in autism spectrum disorder and depression.

#### 3.5.1 Autism spectrum disorder

Li et al. (2023) developed the CRISPR-Human Organoid-Single-Cell RNA Sequencing (CHOOSE) system. They found that intermediate progenitor cells are sensitive to ASD-related gene perturbations, and L2/3 astrocyte-like neurons are more susceptible to ASD-related perturbations—consistent with the co-expression network of ASD risk genes and the characteristics of neuronal impairment in ASD patients. Additionally, they constructed a gene regulatory network for telencephalic developmental trajectories and identified that specific progenitor cells in the dorsal telencephalon (v-RGCs, ccv-RGCs, INPs) are highly enriched. Furthermore, the BAF complex plays a key role in regulating the cell fate of the ventral telencephalon and influencing the progression of ASD, laying the foundation for high-throughput phenotypic characterization of ASD susceptibility genes. Yang K. et al. (2024) identified through whole-exome sequencing that rare variants of IGF1R—a key component of the IGF signaling pathway—are significantly enriched, primarily concentrating in the furin-like repeat domain, fibronectin type III domain, and tyrosine kinase catalytic domain. These variants may disrupt signal transduction and affect cellular functions. Additionally, transcriptomic analysis of brain organoids from ASD patients revealed that the IGF1 and IGF1R modules are associated with early neural development and synaptic function, respectively. Proteomic analysis further showed that IGF1R interacts with multiple ASD susceptibility genes and is regulated by THRA and SCN2A. Based on multi-omics analysis, it was indicated that IGF1R is a core node of the IGF signaling pathway, representing a potential pathogenic mechanism and therapeutic target for ASD.

Wang et al. (2025) found through metabolomic analysis that an increased ratio of *γ*-aminobutyric acid (GABA) to glutamic acid (Glu) is a metabolic characteristic of ASD and can serve as a diagnostic indicator. Multi-omics integrated analysis revealed that GABA metabolic imbalance is a prominent feature of the gut microbiota in children with mild ASD. Additionally, GABA acts as a functional link between the gut microbiota and brain function. Mouse experiments further confirmed that the enrichment of *Escherichia coli* and abnormal GABA metabolism affect social behavior. In summary, the combined application of single-cell sequencing and multi-omics has revealed precise genetic and protein transcriptional changes in ASD patients’ cells at the genetic, transcriptional, proteomic, metabolic, and microbial levels, providing new insights for exploring ASD-related cell states, molecular pathways, pathogenic mechanisms, and therapeutic targets. However, limitations exist in clinical translation and population applicability: although the GABA/Glu ratio identified in metabolomic studies has been validated as a diagnostic marker, it has not been tested in patients with different ASD subtypes to verify its universality; as a potential therapeutic target, IGF1R lacks intervention efficacy experiments at the cellular or animal level, and there is a dearth of preclinical data supporting its safety and effectiveness. Additionally, all studies failed to fully account for ASD genetic heterogeneity, resulting in identified molecular mechanisms and targets that cannot cover ASD patients with diverse etiologies, thus failing to meet the needs of precision diagnosis and treatment.

#### 3.5.2 Depression

Ma et al. (2024) used scRNA-seq and found that the proportion and transcriptome profile of microglia in the prefrontal cortex of patients with depression changed significantly. The abnormal activation or dysfunction of these microglia may trigger neuroinflammation and alterations in synaptic plasticity, which are associated with the pathogenesis of depression. Further analysis revealed differences in multiple microglial subsets between the normal state and depressive state; DEGs are involved in depression-related signaling pathways such as Rap1, cAMP, and TGF-*β*. Among these, YAP1 was identified as a core molecule—it showed significantly increased expression in the prefrontal cortex of depressed patients and had a positive correlation with the severity of depression, making it a promising potential therapeutic target. Hagenberg et al. (2025) integrated immune markers and RNA sequencing data to identify four depressive subgroups: two high-immune-related depressive symptom clusters (characterized by high BMI, severe depression, and elevated immune markers), one mild depressive symptom cluster (with mild depression, slight changes in immune markers, distinguishable by electrocardiogram data, and enriched in brain-related gene sets), and one low-immune-related depressive symptom cluster (with high depression severity but no elevation of immune markers). Structural imaging data showed differences in ventricular volume among different subgroups. After incorporating the predicted cell type proportions into cluster analysis, three subgroups were obtained—one of which had elevated immune markers, and the cell type proportions and cell type-related genes were most prominent in the moderate depressive subgroup. This study reveals the heterogeneity of depression and provides targets for clinical stratification and new therapeutic methods.

Sun et al. (2024) used scRNA-seq analysis and found that the proportion of CD4^+^ naive T cells in patients with major depressive disorder was lower than that in healthy controls, and this abnormality could be reversed after 12 weeks of treatment. DEG analysis showed that the impaired proliferation and differentiation of CD4^+^ naive T cells were associated with depression: decreased expression of the MT-CO1 gene leads to energy metabolism defects, while reduced expression of the JUN and FOS genes affects proliferation. Pseudotime analysis showed that regardless of pre- or post-treatment, responders had a specific population of immune-activated CD4^+^ naive T cells, characterized by enhanced MAPK signaling pathway. Through ATAC-seq analysis, it was found that the MAPK pathway induced by the ETS family upregulates immune responses and cell differentiation in the CD4^+^ naive T cells of responders; moreover, these cells differentiate into regulatory T cells in responders and into Th1 cells in non-responders. It can be seen that this study combines scRNA-seq and ATAC-seq, pointing out that the reduction of CD4^+^ naive T cells can be used as a predictor of poor efficacy of antidepressant treatment, and the activation of CD4^+^ naive T cells mediated by the ETS-MAPK pathway is a characteristic of responders. In summary, integrating single-cell sequencing with multi-omics approaches at genetic, transcriptomic, and proteomic levels reveals precise gene and protein expression changes in cells from depressive patients, providing novel perspectives on cellular transcriptional alterations, immune dysregulation, and therapeutic targets in depression.

### 3.6 Others

In addition to the five major categories of CNS diseases discussed above, single-cell multi-omics technologies have also made significant advances in the research of central nervous system tumors and epilepsy—two disease types with extremely high global clinical burden and unmet medical needs. For glioblastoma, the most lethal primary malignant brain tumor in adults, these technologies have revealed extensive intratumoral cellular heterogeneity and dynamic cellular state plasticity, providing critical insights into the mechanisms underlying tumor recurrence and therapeutic resistance (Ravi et al., 2022). For epilepsy, which affects over 50 million people worldwide with approximately 30% of cases being drug-resistant, single-cell transcriptomics and spatial omics have identified cell type-specific transcriptional alterations and abnormal neural circuit remodeling, facilitating the discovery of novel antiepileptic drug targets (Liu et al., 2024). Further in-depth exploration of these two disease areas will undoubtedly expand the application scope of single-cell multi-omics technologies and promote the development of precision medicine for the full spectrum of CNS disorders.

## 4 Conclusion and future directions

First, the research perspective has advanced from isolated analysis at a single molecular level to multi-dimensional integrated research. By simultaneously integrating data from genomics, transcriptomics, proteomics, metabolomics, and other omics, it systematically decomposes the complex pathological mechanisms of CNS diseases. Relying on single-cell sequencing to accurately identify the abnormal activation or functional disorder of specific cell subtypes, and combining with cross-validation of multi-omics data, it clearly explores the changes of key differential genes, regulatory proteins, and metabolites during disease progression. This further fully reveals the pathogenesis and pathological process of CNS diseases, breaking the limitation that traditional methods are difficult to analyze cell-specific effects and multi-molecular synergistic regulation. Second, the analysis dimension has shifted from averaged signals at the traditional tissue level to precise analysis at the cellular/subcellular level. It can not only deeply capture the cellular heterogeneity in the CNS microenvironment but also, through the integration and screening of cell subtype-specific analysis at the single-cell level and multi-omics data, identify potential disease biomarkers with high specificity and sensitivity from massive molecular information. This effectively solves the problem of insufficient specificity of traditional biomarkers due to interference from average tissue signals, providing reliable indicators for the early diagnosis and disease monitoring of CNS diseases. Third, the research model has expanded from static observation of molecular characteristics to an analytical system with dynamic tracking potential, making it possible to reveal the dynamic evolution process of diseases. At the same time, through the linkage analysis of multi-dimensional data, it accurately locates the molecules and cells that play a key regulatory role in the occurrence and development of diseases, and identifies the core targets for disease treatment. This not only provides a precise direction for the development of targeted treatment plans for CNS diseases but also lays a foundation for the advancement of subsequent clinical stratification and personalized treatment.

In the field of life sciences, the integration of single-cell sequencing and multi-omics technologies has brought revolutionary breakthroughs in analyzing the complex regulatory networks of the CNS. However, they still face multiple challenges and prominent limitations in the research and clinical translation of CNS diseases. On one hand, at the technical level, there are practical issues such as the need to further improve spatial resolution, detection throughput, and the ability to deeply integrate multi-omics data; high application costs of technologies limit the popularization in large-scale clinical samples; there is an urgent need for the promotion of standardized data analysis processes and algorithm innovation; and the difficulty in obtaining human fresh brain tissue samples and strict preservation conditions. In addition, single-cell sequencing is prone to generate noise interference and data sparsity, which reduces the accuracy and reliability of analysis results, and the detection sensitivity for low-abundance molecules such as trace proteins and metabolites is extremely low, making it hard to fully mine key disease-related molecular information. On the other hand, the large differences in dimensions and complex sources of multi-omics data lead to a sharp increase in the difficulty of data integration and interpretation. Moreover, most research results are still in the stage of basic research, lacking large-scale, multi-center clinical verification, making it difficult to directly apply them to the early diagnosis, prognosis evaluation, or treatment plan formulation of diseases. Besides, the inherent heterogeneity of the CNS is extremely significant, with huge differences in cell phenotypes and molecular characteristics across different brain regions and disease stages, so it is difficult to form unified analysis standards and normative reference thresholds.

Currently, in the application research of CNS diseases, the value of single-cell multi-omics technologies has not been fully realized. Most research cases are still limited to single-omics analysis or simple combination of dual-omics, failing to further promote the in-depth integration and linkage analysis of multi-dimensional data such as genomics, transcriptomics, proteomics, and metabolomics. This limitation is also reflected in relevant research papers—especially in studies on CNS infectious diseases. Due to the lack of focused presentation of multi-omics data integration and analysis, the research conclusions are difficult to fully reflect the comprehensive molecular pathological picture of diseases. This not only affects the depth of understanding of disease mechanisms but also restricts the efficiency of translation from research findings to clinical practice to a certain extent, failing to provide more systematic multi-omics evidence support for the precise diagnosis and targeted treatment of CNS diseases. Furthermore, the shortage of professional multi-omics analysis platforms and technical teams targeting CNS diseases, as well as the unavoidable ethical and privacy issues in the collection and analysis of human biological samples, further hinder the standardized promotion and clinical transformation of related technologies.

**Figure 4:**
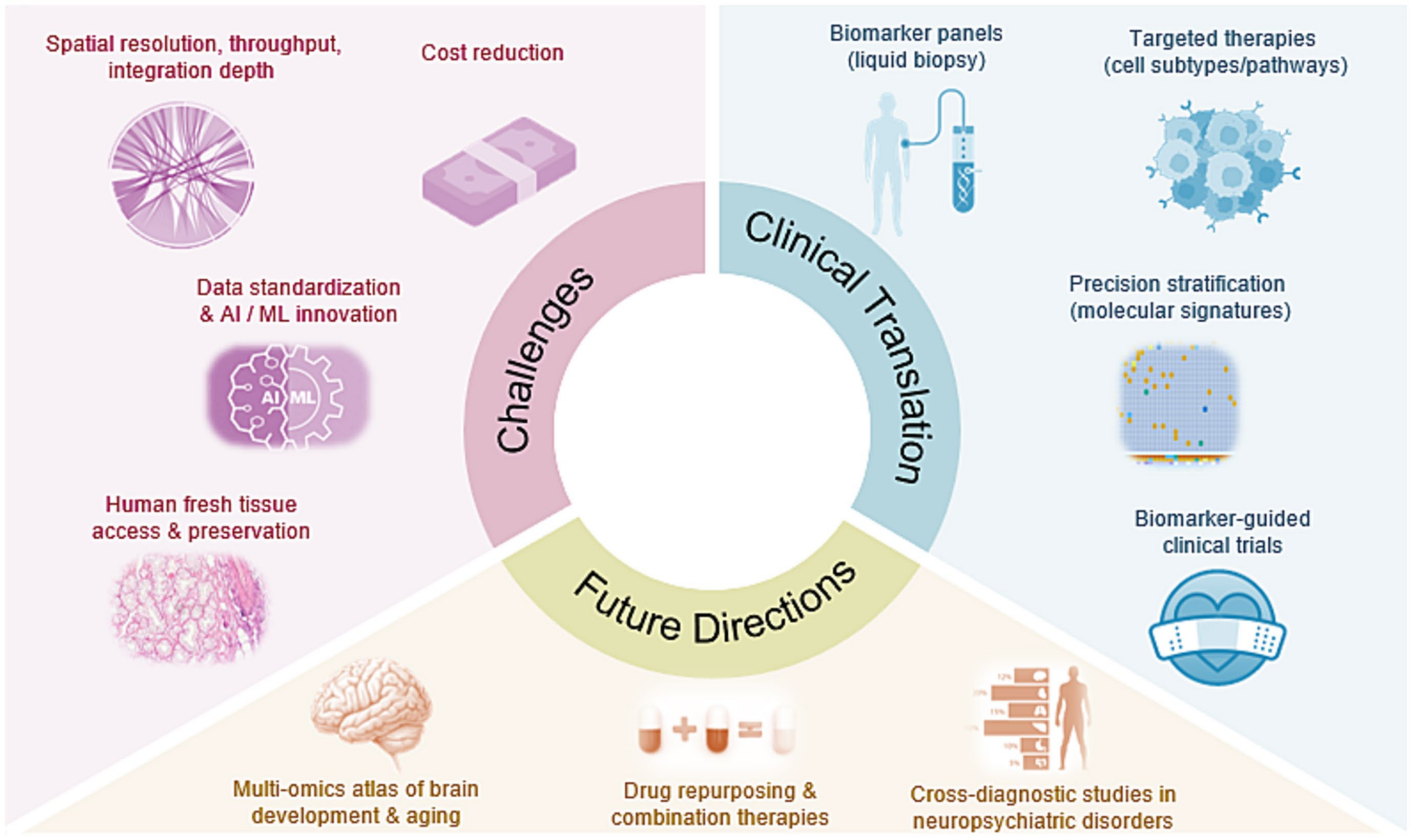
Challenges, clinical translation, and future directions of single-cell multi-omics technologies.

The rapid development of artificial intelligence and machine learning technologies in recent years has provided key tools to break through the above bottlenecks. Through algorithm models for in-depth mining of massive single-cell data, it can not only efficiently identify cell subtypes and analyze gene regulatory networks but also accurately screen potential disease biomarkers, greatly improving the efficiency and accuracy of data processing. On this basis, promoting the translation of technical research findings to clinical practice has become a key breakthrough direction. Specifically, it is necessary to explore the transformation of molecular characteristics mined in research into clinically applicable diagnostic tools (such as biomarker panels based on liquid biopsies), develop new treatment strategies targeting specific cell subsets or pathogenic pathways, and establish a precise patient classification system based on molecular characteristics. At the same time, it is necessary to face the challenges in clinical trial design (especially the complexity of clinical trials guided by biomarkers in terms of enrollment criteria setting and efficacy evaluation index establishment). In the future, single-cell multi-omics technologies will focus on mapping the systematic atlas of human brain development and aging processes, helping to analyze the transdiagnostic molecular mechanisms of neuropsychiatric diseases, and guiding drug repurposing research and combination therapy development based on multi-dimensional omics data. This interdisciplinary approach will not only continuously promote the progress of basic research on the CNS but also provide important references for the development of clinical diagnosis and treatment plans in the future. It is expected to realize a comprehensive innovation in the diagnosis and treatment of the CNS, bringing broader development space for the research and clinical diagnosis and treatment of CNS diseases (Figure 4).

Based on the above discussion, the research group is currently exploring the application of Stereo-cell—an analytical tool for specific cell types in the CNS—in cerebrospinal fluid (CSF) analysis. As a biological fluid in direct contact with the CNS, changes in CSF composition are closely related to the physiological state of nerve cells and disease progression, making it a “window” reflecting the function and pathological state of the CNS. The characteristics of Stereo-cell technology endow it with unique advantages in CSF analysis. First, Stereo-cell technology breaks the limitation of traditional cell analysis on cell size, efficiently capturing and analyzing nerve cells of different sizes and pathologically abnormal cells in CSF, thus avoiding the loss of sample information. Second, it meets the needs of both small-sample fine analysis and large-scale clinical cohort research, enabling efficient processing of samples containing 100,000 to millions of cells. Third, it supports combination with a variety of cell staining technologies to realize the visualization of cell morphology and spatial location information, facilitating the understanding of the association between cell functions and disease phenotypes. Fourth, through its multi-omics integration capability, it constructs a “gene-protein-metabolism” map. Combined with the dynamic tracking function of on-chip cell culture, it makes up for the limitation of traditional static sequencing. At the same time, it can capture extracellular vesicles, providing support for analyzing the dynamic process of diseases, intercellular communication, and exploring biomarkers. Collectively, these features position Stereo-cell as a powerful tool for advancing CNS research from mechanistic insights to clinical translation, offering promising avenues for improved diagnosis, monitoring, and therapy of CNS disorders.
